# Airway-pressure challenge during spontaneous breathing: a conceptual framework for interpreting hemodynamic vulnerability to positive-pressure ventilation in dogs and cats

**DOI:** 10.3389/fvets.2026.1859636

**Published:** 2026-06-08

**Authors:** Shotaro Nagahama

**Affiliations:** 1 JAVA Incorporated Association, Tokyo, Japan; 2 Department of Veterinary Anesthesia, Veterinary Teaching Hospital, Faculty of Applied Biological Sciences, Gifu University, Gifu, Japan

**Keywords:** arterial pressure, cardiopulmonary interactions, cats, dogs, hemodynamic vulnerability, positive-pressure ventilation, right ventricular afterload, spontaneous breathing

## Abstract

Positive-pressure ventilation can precipitate an immediate fall in arterial blood pressure during general anesthesia, but this response is usually recognized only after controlled ventilation has already been initiated and is then interpreted retrospectively. We propose a different framework for dogs and cats: a brief airway-pressure challenge during spontaneous breathing may serve as a deliberate, reversible bedside probe of hemodynamic vulnerability to subsequent positive-pressure ventilation. The aim of this article is to articulate an interpretive, hypothesis-generating framework rather than to provide a validated protocol or an immediately deployable clinical test. In this view, the immediate arterial pressure response is interpreted not as a specific diagnostic sign or a simple test of circulating volume adequacy, but as an integrated cardiopulmonary stress response. Its meaning may be shaped by preload sensitivity, right ventricular afterload sensitivity, tricuspid regurgitation, pulmonary vascular burden, anesthetic state, myocardial reserve, and respiratory mechanics. Framing the maneuver in this way shifts attention from incidental observation to proactive interpretation and may help clinicians anticipate whether positive-pressure exposure is likely to be well tolerated or to warrant greater caution, closer monitoring, and more deliberate circulatory preparation. The framework also gives additional conceptual value to preserving spontaneous breathing before mandatory controlled ventilation, because this phase provides an interpretive window before the circulation is fully exposed to sustained positive-pressure ventilation. At the same time, this concept is not presented as a validated diagnostic test or treatment algorithm. Nor is it intended to recommend routine clinical implementation of the airway-pressure challenge as a ventilation method prior to technical standardization and prospective validation. Its clinical interpretation is context dependent and is best suited to settings in which immediate arterial pressure changes can be followed continuously, preferably with invasive arterial pressure monitoring. By reframing airway-pressure–associated hypotension as a structured challenge rather than a purely incidental event, this conceptual model provides a basis for future physiologic validation while supporting more deliberate bedside interpretation of cardiopulmonary vulnerability in veterinary anesthesia.

## 1 Introduction

Positive-pressure ventilation can produce an immediate decrease in arterial blood pressure during general anesthesia. In clinical practice, this response is familiar, but it is usually encountered only after controlled ventilation has already been initiated. At that point, the blood pressure decrease is interpreted retrospectively in light of anesthetic depth, vascular tone, circulating volume, cardiac function, airway pressure, and ventilatory settings, and is therefore treated more as an accompanying event than as a deliberately examined physiologic signal (1, 2).

This conventional posture is understandable. The hemodynamic effects of positive-pressure ventilation are inherently multifactorial, reflecting changes in pleural pressure and transpulmonary pressure that influence venous return, right ventricular afterload, pulmonary vascular loading, and the downstream transmission of right-sided output to the systemic circulation (1, 3, 4). At the same time, bedside hemodynamic assessment has increasingly emphasized transient, defined perturbations rather than static variables alone. In awake or otherwise cooperative spontaneously breathing patients, maneuvers such as the Valsalva maneuver and deep inspiration have been studied as reversible tests of fluid responsiveness, whereas in mechanically ventilated patients tidal-volume challenge and brief PEEP elevation have been used for similar purposes (5–8).

We therefore propose that a brief airway-pressure challenge during spontaneous breathing may be viewed as a deliberate, reversible hemodynamic maneuver. In this framing, the response to transient airway-pressure loading is not reduced to a simple test of circulating volume adequacy, nor treated as a specific sign of any single abnormality. Instead, it is interpreted as a functional cardiopulmonary stress response that may help reveal how vulnerable a given patient is to subsequent positive-pressure ventilation. Conceptually, this aligns with existing functional hemodynamic maneuvers, but it is directed toward a different bedside question: not simply whether stroke volume may increase after fluid administration, but whether the circulation appears fragile when exposed to positive airway pressure before sustained controlled ventilation is imposed (5, 6, 8).

This perspective is especially relevant when spontaneous breathing is being maintained, because spontaneous breathing preserves a pre-transition state before the circulation is fully conditioned by sustained controlled ventilation. The aim of this article is therefore not to argue that clinicians are unaware of the multifactorial nature of ventilation-associated hypotension, nor to prescribe a standardized maneuver or claim immediate clinical validation, but to define a conceptual framework that clarifies how this familiar response may be interpreted and studied prospectively.

## 2 From incidental observation to deliberate challenge

In routine anesthetic practice, the hemodynamic effects of positive-pressure ventilation are usually recognized only after controlled ventilation has already begun. A decrease in arterial blood pressure is then interpreted as part of the newly imposed ventilatory state, together with changes in anesthetic depth, carbon dioxide, vascular tone, and any interventions that follow in response. In this familiar sequence, the response may be informative, but it is not intentionally sought (1, 2).

This timing matters. Once controlled ventilation is established, the observed hemodynamic response is already embedded within a broader physiologic transition. Airway pressure is no longer a brief perturbation but part of an ongoing condition, and interpretation becomes less clean because several changes have occurred at once. In mechanistically oriented studies, respiratory maneuvers gain diagnostic value precisely because they are short, defined, and reversible; once the perturbation becomes prolonged, separating the contribution of the maneuver itself from that of the evolving clinical state becomes more difficult (5–8).

We propose a different starting point. Rather than waiting for controlled ventilation to reveal instability incidentally, a brief airway-pressure challenge during spontaneous breathing may be used deliberately as a limited and reversible hemodynamic perturbation. The aim is not to reproduce the full physiologic state of sustained controlled ventilation, but to introduce a short-lived stress that may help disclose how vulnerable the circulation is to subsequent positive-pressure exposure. In this sense, the maneuver is proactive rather than reactive: it is performed to gain interpretive information before sustained positive-pressure exposure occurs, not merely to explain hypotension after it appears. This logic parallels the established use of transient respiratory maneuvers as functional hemodynamic tests in other settings (5, 6, 8, 9).

**Figure 1:**
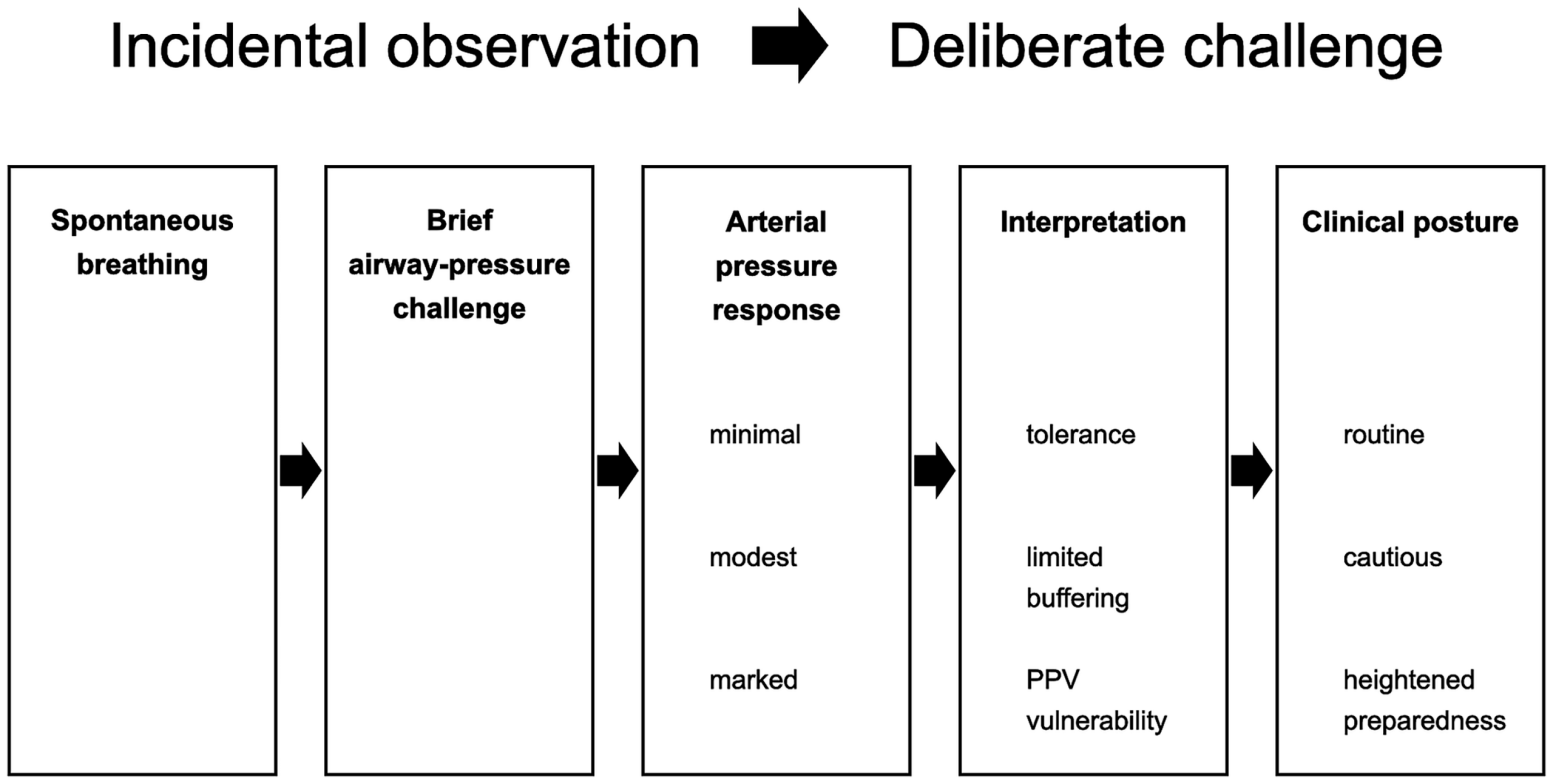
The airway-pressure challenge during spontaneous breathing as a deliberate probe of hemodynamic vulnerability to positive-pressure ventilation (PPV). This conceptual schematic illustrates a shift from incidental observation to deliberate challenge. During spontaneous breathing, a brief airway-pressure challenge is applied before sustained positive-pressure ventilation, and the immediate arterial pressure response is interpreted as a graded indicator of tolerance, limited buffering capacity, or vulnerability to subsequent PPV. The purpose of the maneuver is not to establish a specific diagnosis, but to support interpretation and transition planning. This framework is best suited to settings in which immediate arterial pressure changes can be followed continuously, preferably with invasive arterial pressure monitoring. The figure is intended as a conceptual schematic and should not be interpreted as a clinical algorithm or recommended ventilatory protocol.

This shift changes the meaning of the response. A blood pressure decrease during transient airway-pressure loading is no longer viewed simply as an undesirable side effect of ventilation, nor as an isolated clue pointing toward a single diagnosis. Instead, it becomes the output of a defined bedside maneuver. What matters is not only that blood pressure changes, but that the change occurs in response to a known and limited perturbation introduced before the patient has been fully exposed to sustained positive-pressure ventilation. The proposed novelty therefore lies less in any new mechanism than in reframing a familiar cardiopulmonary interaction as an intentional bedside challenge (Figure 1).

## 3 The airway-pressure challenge as a functional hemodynamic probe

The conceptual value of an airway-pressure challenge lies in viewing it not simply as a brief ventilatory intervention, but as a functional hemodynamic probe. Functional hemodynamic assessment derives information not only from absolute values, but from the circulation’s response to a defined perturbation in loading conditions. In awake or otherwise cooperative spontaneously breathing patients, the Valsalva maneuver and deep inspiration have been shown to improve interpretation of arterial pressure or flow changes, while in mechanically ventilated patients tidal-volume challenge and short PEEP elevation have been used for a similar purpose (5–8). These precedents support the idea that a transient respiratory perturbation can serve as an informative cardiovascular probe.

This framing is important because the maneuver should not be reduced to a crude test of circulating volume adequacy. A blood pressure decrease during airway-pressure loading may be compatible with preload dependence, but it is not specific for low circulating volume and should not be interpreted as such. Positive-pressure breathing simultaneously affects pleural pressure, transpulmonary pressure, venous return, and right ventricular afterload, and dynamic indices obtained during respiratory maneuvers may reflect afterload effects as well as preload dependence (1, 2, 4). The maneuver is therefore better understood as a probe of hemodynamic tolerance to positive-pressure exposure than as a direct assay of volume status.

Its purpose is not to diagnose a single mechanism, but to reveal whether even a brief, limited increase in airway pressure is associated with immediate hemodynamic instability. The more pronounced the response, the more cautiously the clinician may wish to interpret the patient’s tolerance for sustained positive-pressure ventilation. The response should therefore be interpreted relationally rather than categorically: the key question is not simply whether blood pressure decreases, but how readily it decreases in relation to a known airway-pressure perturbation.

Because the challenge is intended as a functional probe, its meaning depends on boundedness and reversibility. It should be conceptualized as a brief perturbation rather than as an open-ended progression into full controlled ventilation. At present, it is best regarded not as a standardized quantitative instrument, but as a structured physiologic maneuver whose output is context dependent. That caution is consistent with both human and veterinary dynamic-monitoring literature. In dogs under general anesthesia and mechanical ventilation, pulse pressure variation and related dynamic indices can predict fluid responsiveness with useful accuracy, but their interpretation remains conditional on ventilatory conditions and physiologic context (10–12). The present concept extends that logic to a different bedside purpose: using a brief airway-pressure perturbation during spontaneous breathing to explore vulnerability to subsequent positive-pressure ventilation. At this stage, the concept is intentionally framed at the level of physiologic interpretation rather than as a fixed technical protocol, because the magnitude, duration, and delivery conditions of the challenge remain matters for future standardization and validation.

## 4 A multifactorial framework for interpreting the response

The hemodynamic meaning of an airway-pressure challenge depends on the fact that the observed blood pressure response is not generated by a single mechanism. Rather, it emerges from the combined behavior of the venous system, the right heart, the pulmonary circulation, the left heart, vascular tone, and the anesthetized respiratory system. Modern physiologic descriptions of positive-pressure ventilation distinguish the effects of pleural-pressure changes on venous return from the effects of transpulmonary-pressure changes on right ventricular afterload and pulmonary vascular loading (1, 2, 13). Accordingly, the challenge should not be interpreted through a unitary lens.

One important component is the effect of airway pressure on venous return. A transient rise in pleural pressure can reduce the pressure gradient for systemic venous return and thereby decrease right ventricular filling. In some patients this effect may be modest, whereas in others even a brief increase in airway pressure may cause a noticeable reduction in forward flow. This does not necessarily imply absolute hypovolemia. Rather, it may indicate that the circulation is operating in a state in which cardiac output remains sensitive to perturbations in preload (1, 2, 14).

A second major component is the effect of airway pressure on right ventricular ejection. Increased airway and transpulmonary pressures may alter pulmonary vascular loading conditions and increase the effective afterload faced by the right ventricle. Because the right ventricle is relatively sensitive to acute afterload elevation, positive-pressure ventilation can unmask or worsen right-ventricular dysfunction even when overt right-heart failure was not previously recognized (13, 15–17). Thus, a blood pressure fall during airway-pressure loading may reflect not only impaired filling, but also impaired right-sided adaptation to positive-pressure stress.

The response may be amplified further when tricuspid regurgitation is present. Significant tricuspid regurgitation imposes a volume load on the right ventricle, is linked to right-ventricular remodeling and dysfunction, and becomes clinically more important as right-ventricular adaptation worsens or pulmonary vascular load increases (18–20). From the standpoint of the present framework, this means that the same airway-pressure challenge may have different consequences depending on whether the right ventricle can preserve effective pulmonary forward flow under altered loading conditions.

A third major component is the anesthetic state itself. General anesthesia can reduce vascular tone, blunt compensatory autonomic responses, and diminish myocardial reserve. Under these conditions, the circulation may tolerate baseline spontaneous breathing while remaining poorly buffered against a transient rise in airway pressure. Prospective peri-induction work has described anesthesia induction with the initiation of positive-pressure ventilation as a vulnerable phase and has shown measurable reductions in right-ventricular longitudinal function after induction and IPPV, even when global right-ventricular output appears preserved (17, 21).

Respiratory mechanics also shape the meaning of the challenge. The same nominal airway pressure does not necessarily produce the same intrathoracic or transpulmonary effect across patients. Lung compliance, chest wall compliance, abdominal pressure, body position, and ongoing inspiratory effort all influence how airway pressure is transmitted to the cardiovascular system. Consequently, similar applied pressures may yield very different hemodynamic responses, not only because circulatory reserve differs, but also because mechanical coupling differs (1, 2, 13).

Finally, the blood pressure response reflects downstream interactions between the right and left sides of the circulation. A transient reduction in right-ventricular forward output does not translate into systemic arterial pressure in a uniform way; its expression depends on timing, vascular tone, and the buffering capacity of the systemic circulation. Taken together, these considerations support a multifactorial interpretation of airway-pressure challenge responses: the observed change in arterial pressure may reflect varying contributions from preload sensitivity, right-ventricular afterload sensitivity, tricuspid regurgitation, pulmonary vascular burden, anesthetic vasodilation, reduced myocardial reserve, and patient-specific respiratory mechanics.

## 5 Clinical implications of the airway-pressure challenge

If a brief airway-pressure challenge during spontaneous breathing is interpreted as a functional probe of hemodynamic vulnerability, its main clinical value lies not in producing a diagnosis, but in informing how the patient’s cardiopulmonary vulnerability is understood at the bedside. The challenge does not tell the clinician whether any subsequent intervention is categorically safe or unsafe. Rather, it helps identify whether the patient appears hemodynamically tolerant of positive-pressure exposure or whether greater caution, closer monitoring, and more deliberate circulatory support may be warranted. In practical terms, the framework asks the clinician not to assign a single diagnosis to the response, but to judge whether the observed arterial pressure change is negligible, moderate, or pronounced relative to the applied perturbation, and then to interpret that response as evidence of greater or lesser cardiopulmonary reserve under positive-pressure stress. This way of thinking fits with contemporary perioperative hemodynamic guidance, which increasingly emphasizes anticipation, context, and multivariable interpretation over reflexive single-cause reasoning (14, 21, 22).

A first implication is that positive-pressure exposure may be reframed as a graded hemodynamic event rather than as a purely respiratory adjustment. In routine practice, the decision to initiate controlled ventilation is usually driven by gas exchange, carbon dioxide control, procedural requirements, or because spontaneous breathing no longer appears sufficient to meet respiratory demands. These indications remain valid. However, when an airway-pressure challenge has already suggested limited tolerance to positive-pressure loading, the clinician may interpret subsequent positive-pressure exposure as a foreseeable cardiovascular stress rather than as a neutral technical step (17, 21).

A second implication is that the response to challenge may help shape how controlled ventilation is introduced. If transient airway-pressure loading produces little hemodynamic effect, the patient may appear relatively tolerant of positive-pressure exposure under the current anesthetic conditions. If the response is pronounced, the clinician may wish to minimize the circulatory cost of transition by using the least hemodynamically stressful ventilatory conditions compatible with the respiratory goal and by avoiding abrupt shifts in preload, systemic pressure, or pulmonary vascular loading. That principle is especially well developed in perioperative right-heart and pulmonary-hypertension guidance, but its logic is broadly relevant whenever positive-pressure ventilation risks stressing the right ventricle (23, 24).

A third implication concerns circulatory preparation. A marked blood pressure decrease during a brief airway-pressure challenge may suggest that sustained controlled ventilation could reveal or amplify limited cardiopulmonary reserve. This does not automatically indicate a need for volume administration, nor does it mandate any single intervention. Rather, it encourages anticipatory reasoning: the clinician may consider whether the circulation appears primarily vulnerable to reduced preload, right-sided loading conditions, anesthetic-related vasodilation, or limited myocardial reserve. This posture is also consistent with recent perioperative and veterinary literature, which argues against equating hemodynamic instability with automatic fluid administration and supports more selective use of fluids and vasoactive therapy (14, 21, 25, 26).

This perspective also supports the clinical value of preserving spontaneous breathing, when appropriate, before mandatory controlled ventilation. In the present framework, spontaneous breathing is valuable not only because it may reduce exposure to sustained positive-pressure loading, but also because it preserves an interpretive phase during which the circulation can be observed before the full hemodynamic consequences of controlled ventilation are imposed. In practical terms, this framework is likely to be most informative when immediate arterial pressure responses can be followed continuously, making invasive arterial pressure monitoring the preferred monitoring context for clinical interpretation of the challenge. The challenge response is therefore useful less as a binary gatekeeper than as a way to organize expectation: a minimal response may suggest relative tolerance, whereas a rapid or exaggerated response may justify closer attention to monitoring, ventilatory settings, and readiness for circulatory support during the subsequent transition.

Importantly, the absence of a dramatic blood pressure response should not be overinterpreted. A patient may appear tolerant of brief airway-pressure loading and still develop hemodynamic instability once controlled ventilation becomes prolonged, carbon dioxide changes accumulate, or anesthetic conditions evolve. Conversely, an early response may be informative even if the eventual transition is accomplished successfully, because it identifies a circulation in which tolerance depends on careful ventilatory and hemodynamic management rather than on robust intrinsic reserve (14, 17, 21).

## 6 Limitations and boundaries of interpretation

The proposed airway-pressure challenge is intended as a conceptual bedside probe, not as a validated diagnostic test. Its usefulness depends on interpretation, context, and restraint. Any clinical value it may offer is therefore inseparable from a clear understanding of its limitations. The maneuver is best regarded as a structured way to reveal physiologic vulnerability, not as a means of assigning a definitive mechanism or generating a binary answer about whether controlled ventilation will or will not be tolerated (14, 21).

A first limitation is the problem of standardization. Even if the same nominal airway pressure is applied, the physiologic meaning of that pressure is not constant across patients. The transmission of airway pressure to intrathoracic structures is influenced by lung compliance, chest wall compliance, abdominal pressure, body position, inspiratory effort, and airway resistance. A given airway-pressure challenge therefore cannot be assumed to represent the same cardiovascular perturbation in every patient (1, 2, 13).

A second limitation is that arterial blood pressure is an imperfect readout of the underlying hemodynamic response. Blood pressure is clinically accessible and therefore attractive, but it is shaped not only by forward flow but also by vascular tone, arterial compliance, and altered compensatory conditions under anesthesia. A marked reduction in stroke volume may be incompletely reflected in mean arterial pressure, while an exaggerated pressure response may occur in a circulation with limited buffering capacity even when the primary perturbation is modest. For this reason, the challenge should not be conceptualized as a pure blood-pressure test. Rather, blood pressure should be understood as one practical but partial output of a broader cardiopulmonary response. This limitation is consistent with both perioperative consensus statements and veterinary dynamic-monitoring work, which caution against single-variable interpretation of circulatory status (10, 11, 21, 22).

In addition, the practical interpretability of the challenge depends strongly on how arterial pressure is monitored. Because the proposed maneuver is brief and intended to detect immediate hemodynamic responses, invasive arterial pressure monitoring is likely to provide the most suitable readout. Intermittent noninvasive blood pressure measurements may fail to capture the timing, magnitude, or transient structure of the response and are therefore less well suited to this framework. Accordingly, the present concept is best understood as being most applicable in settings where continuous invasive arterial pressure monitoring is available.

A related limitation is lack of diagnostic specificity. A fall in arterial pressure during transient airway-pressure loading does not identify a single mechanism. It may reflect preload sensitivity, impaired right-ventricular adaptation, pulmonary vascular burden, tricuspid regurgitation, anesthetic vasodilation, reduced myocardial reserve, and altered respiratory mechanics, or several of these together. Its role is to suggest vulnerability domains, not to establish a solitary diagnosis (1, 14, 18).

The maneuver also has temporal limitations. A brief airway-pressure challenge can reveal immediate hemodynamic fragility, but it does not reproduce all of the conditions associated with sustained controlled ventilation. Once controlled ventilation is maintained over time, carbon dioxide may change further, anesthetic conditions may evolve, and cumulative ventilatory effects may emerge that were not evident during a short perturbation. Accordingly, a limited or absent response to challenge should not be taken to guarantee later stability (14, 17).

Another boundary concerns safety and clinical appropriateness. The concept presupposes that the challenge remains brief, reversible, and physiologically bounded. It is not intended for indiscriminate use in all patients, nor should it be applied in a way that introduces avoidable instability. In patients already showing marked hemodynamic compromise, severe hypoxemia, major airway uncertainty, profound anesthetic instability, pulmonary hypertension, or evident right-heart vulnerability, the conceptual appeal of the maneuver may be outweighed by its immediate practical risk (23, 24).

Finally, this concept remains provisional. The present article proposes an interpretive framework, not a validated protocol. It does not establish the optimal magnitude, duration, or technical form of the challenge, nor does it define which hemodynamic outputs should be preferred for bedside interpretation. It also does not claim outcome evidence showing that use of this framework improves management or reduces complications. Accordingly, the airway-pressure challenge should not yet be understood as a protocolized ventilatory maneuver or as a method recommended for routine clinical implementation. At this stage, its value lies in organizing a clinically recognizable phenomenon into a form that may be discussed, studied, and refined, rather than in presenting it as a settled technique (25–27).

Future work should determine the reproducibility, technical standardization, and species-specific performance of the airway-pressure challenge in dogs and cats. Such studies should examine the magnitude and duration of the perturbation, the influence of respiratory mechanics and underlying cardiopulmonary disease, the most informative hemodynamic outputs, and the relationship of the response to other monitoring variables. Prospective validation will also be needed to define how consistently the challenge identifies clinically relevant vulnerability to positive-pressure exposure and whether it improves interpretation or management in practice.

## 7 Conclusion

A decrease in arterial blood pressure during positive-pressure ventilation is a familiar phenomenon in anesthetic practice, but it is usually encountered as an accompanying event after controlled ventilation has already begun. In this article, we propose a different framing: a brief airway-pressure challenge during spontaneous breathing may be interpreted not merely as an incidental cause of hypotension, but as a deliberate and reversible bedside maneuver that helps reveal hemodynamic vulnerability to subsequent positive-pressure ventilation.

The value of this perspective lies in shifting attention from passive recognition to proactive interpretation. Rather than asking only why blood pressure fell after controlled ventilation was imposed, the clinician may ask whether a limited, transient airway-pressure load already discloses restricted tolerance to positive-pressure exposure. In this view, the observed response is not reduced to a simple indicator of circulating volume inadequacy, nor assigned to any single mechanism. Instead, it is understood as an integrated cardiopulmonary stress response shaped by preload sensitivity, right-ventricular afterload sensitivity, pulmonary vascular conditions, tricuspid regurgitation, anesthetic state, myocardial reserve, and patient-specific respiratory mechanics.

This framework also gives additional conceptual value to the preservation of spontaneous breathing before mandatory controlled ventilation. Spontaneous breathing maintains an interpretive window in which cardiopulmonary reserve can be probed before the patient is fully committed to sustained positive-pressure ventilation. A brief airway-pressure challenge, when clinically appropriate, may therefore help make the hemodynamic consequences of positive-pressure exposure more anticipated, more interpretable, and less purely reactive. At the same time, this concept should be understood within clear limits: the proposed maneuver is not a validated diagnostic test, does not establish a specific mechanism, and cannot by itself determine how controlled ventilation should be managed in every case. Its present role is more modest but still potentially useful: to organize a familiar yet often tacitly interpreted phenomenon into an explicit bedside framework that may support physiologic reasoning during anesthetic care.

Accordingly, we suggest that brief airway-pressure loading during spontaneous breathing be considered as a functional hemodynamic probe of ventilation-associated vulnerability in dogs and cats. By reframing the response as a structured challenge rather than a purely incidental event, this conceptual model may offer a useful foundation for future physiologic study, clinical refinement, and more deliberate bedside interpretation of cardiopulmonary vulnerability in veterinary anesthesia.
